# Non-steric-zipper models for pathogenic α-synuclein conformers

**DOI:** 10.1063/1.5023460

**Published:** 2018-05-01

**Authors:** Brock Schuman, Amy Won, Koroboshka Brand-Arzamendi, James B. Koprich, Xiao-Yan Wen, Patrick A. Howson, Jonathan M. Brotchie, Christopher M. Yip

**Affiliations:** 1 St. Michael's Hospital, 30 Bond Street, Toronto, Ontario M5B 1W8, Canada; 2 Institute of Biomaterials and Biomedical Engineering, University of Toronto, Toronto, Ontario M5S 3G9, Canada; 3 Krembil Institute, Toronto Western Hospital, University Health Network, Toronto, Ontario M5T 2S8, Canada

## Abstract

Parkinson's disease neurodegenerative brain tissue exhibits two biophysically distinct α-synuclein fiber isoforms—single stranded fibers that appear to be steric-zippers and double-stranded fibers with an undetermined structure. Herein, we describe a β-helical homology model of α-synuclein that exhibits stability in probabilistic and Monte Carlo simulations as a candidate for stable prional dimer conformers in equilibrium with double-stranded fibers and cytotoxic pore assemblies. Molecular models of β-helical pore assemblies are consistent with α-synuclein^A53T^ transfected rat immunofluorescence epitope maps. Atomic force microscopy reveals that α-synuclein peptides aggregate into anisotropic fibrils lacking the density or circumference of a steric-zipper. Moreover, fibrillation was blocked by mutations designed to hinder β-helical but not steric-zipper conformations. β-helical species provide a structural basis for previously described biophysical properties that are incompatible with a steric-zipper, provide pathogenic mechanisms for familial human α-synuclein mutations, and offer a direct cytotoxic target for therapeutic development.

## INTRODUCTION

α-Synuclein (αSyn) aggregation is a pathological hallmark of both familial and idiopathic Parkinson's disease (PD).^1,2^ Predating αSyn genetic characterization, the protein was known as the Non-Amyloid-Component (NAC) of Lewy bodies, an acronym still used to describe the central aggregation-prone domain of αSyn. Discoveries of αSyn mutations associated with early onset PD^3^ cemented its causative role; however, it is still not clear what that role is or how to reverse it. In its monomeric, synaptic vesicle-bound form, the physiological roles of αSyn remain somewhat elusive but are related to synaptic efflux^4^ and vesicular transport^5^ which is severely retarded in knock-down studies.^6^ In PD, αSyn aggregates into higher-order species including dimers, annular pores, and fibers. These pathogenic conformations play pivotal roles in PD initiation, neurotoxicity, and transcellular propagation. Characterization of these pathogenic structures has been hampered by the complex aggregation behavior and innate structural plasticity of αSyn. While high-resolution nuclear magnetic resonance (NMR) datasets of the labile monomeric micelle-bound αSyn conformers^7,8^ and a single-stranded Greek key steric-zipper (GK) decamer have been obtained,^9^ they have not proven to be sufficient to describe a direct mechanism of neurodegeneration.

Pathogenic conformations of αSyn are driven by the central NAC domain, with residues 71–82 essential for fibrillogenesis.^10^ This region normally binds synaptic vesicles as an amphipathic α-helix (residues 61–95) and is disordered in solution. The NAC domain is a discontinuous β-sheet in pathological conformations, flanked by an N-terminal (NT), an amphipathic helix (residues 1–35), and a labile acidic C-terminal (CT) tail (residues 95–140). The PD-associated familial mutations A30P, E46K, H50Q, G51D, and A53T reside in an ambiguous boundary between the NT and NAC domains, as does Y39, which has been shown to form covalent Y39-Y39 di-tyrosine dimers and inter-fiber crosslinks both *in vitro*^11^ and *in situ.*^12^

Electron microscopy (EM) and AFM experiments revealed that αSyn fibrils adopt variable morphologies with two very biophysically distinct species (reviewed in the study by Bousset *et al.*^13^) existing as either single- or double-stranded cylinders or double-stranded ribbons (dsRibbons), the latter of which do not appear to be comprised of individual cylinder protofilaments but rather a totally unique conformation. Compared to dsRibbons, cylinders *in vitro* require consistent high salt concentrations, are sensitive to temperature, and are more transient species that degrade to seed dsRibbons.^13^ Conversely, dsRibbons appear to be significantly more robust and do not degrade to seed cylinders.^13^ dsRibbons routinely present in EM as two ∼4 × 5.5 nm strands separated by ∼1.4 nm^14^ and in AFM as 5.2–6.6 nm high ribbons.^15,16^ Such an ∼1.4 nm gap is not observed for cylindrical protofilament dimers. While straight dsRibbons have been observed,^17,18^ dsRibbons more often exhibit a helical periodicity that is regular within a single fibril but variable between fibrils. Both left-handed helices (LHHs) with an 81–141 nm pitch^17,19^ and right-handed helices (RHHs) with an ∼45 nm pitch have been reported for wild-type αSyn.^20^ Structural characterization by fiber diffraction, infrared spectroscopy, and solid-state NMR (ssNMR)^14,21,22^ has revealed the fibrillar NAC to be comprised of discontinuous, parallel β-sheets that hydrogen bond parallel to the fiber axis. The exact boundaries of the sheets appear as a variable by ssNMR, beginning in the range of residues 35–41 and ending in the range of residues 92–96, indicating substantial plasticity even in this relatively ordered conformation.

**FIG. 1.**
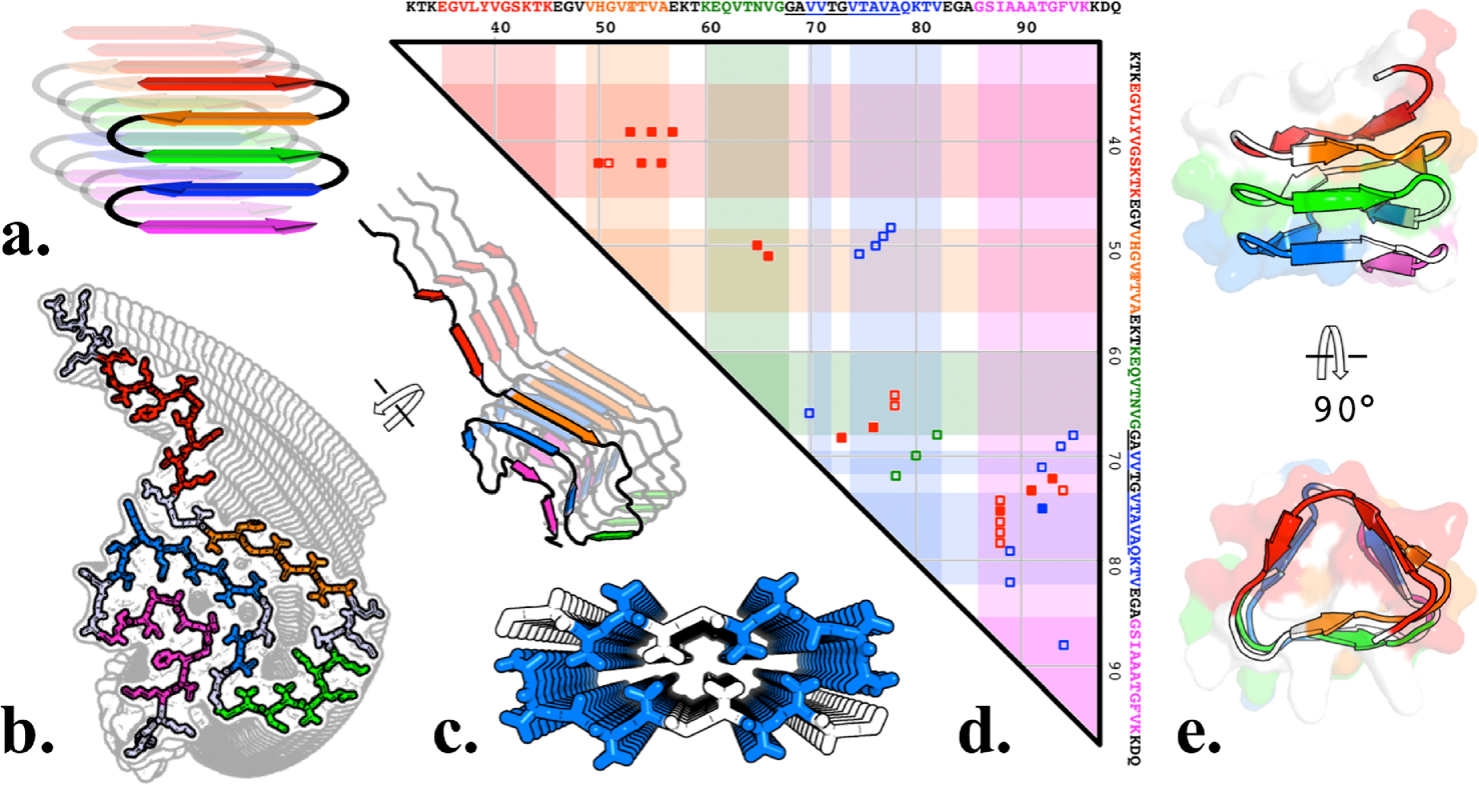
Steric-zipper disparities. (a) “Five-layer-β-sandwich” proposed steric-zipper, with each of the five β-sheets observed to interact by NMR colored analogously throughout the figure. (b) GK NAC 30mer displaying inherent right-handedness and secondary structure to display the steric-zipper configuration. (c) “NACore” twofold small peptide. (d) Unambiguous <6 Å distance restraints of two-stranded fiber residues^5^ (red markers) are predominantly satisfied (filled red markers) by our β-helix model and (e) <5% of the observed distance restraints are coincident with those of GK (blue markers) and none with NACore (green markers), indicating that neither GK nor NACore structure are representative of a two-stranded fiber.

To reflect the NMR-derived β-sheet boundaries and observed intrasheet interaction between residues, a model was proposed in which all β-sheets are intermolecular in a serpentine steric-zipper [Fig. 1(a)^14^]. Two incompatible atomic models of αSyn steric zippers have been published: an ssNMR structure of the intact αSyn GK ssFiber decamer, which was not suggested to be representative of dsRibbons [Fig. 1(b), Protein Data Bank (PDB) identifier 2N0A^9^], and a microcrystal electron diffraction structure of NAC residues 68–79 [Fig. 1(c), PDB 4R0W^23^]. In the present work, we present models in which the dsRibbons exist in equilibrium with other pathological conformations, which are distinct from the denser GK cylinder. These models were designed to provide rationale for clinical and biophysical observations and may be of use to guide *ab initio* model design for high-resolution experimental data and to design disease-modifying therapeutics.

### Two-stranded steric-zipper inconsistencies

**FIG. 2.**
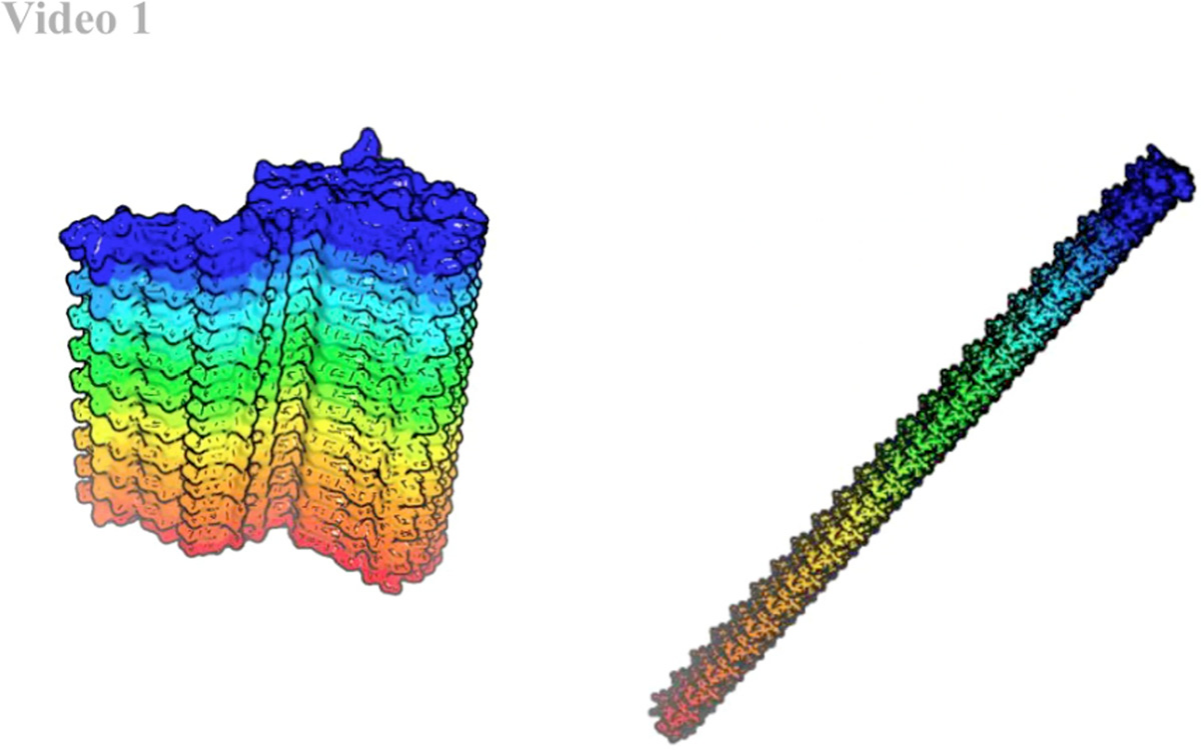
Fiber handedness. Steric-zippers such as GK are comparatively rigid and can only produce straight or mildly right-handed helices. β-helical subunits, on the other hand, can produce left- to right-handed helices with variable periodicity with moderate changes to their intramolecular interfaces. Multimedia view: https://doi.org/10.1063/1.5023460.110.1063/1.5023460.1

While αSyn cylinders are likely steric-zippers, several lines of evidence suggest that αSyn dsRibbons are not. First, dsRibbons are much more susceptible to proteolysis than cylinders,^13^ suggesting a less dense conformation with more solvent-accessible cleavage sites [Fig. S1(b)]. Second, NACore and GK inter-residue distances are mutually exclusive to the ssNMR distance restraints obtained for hydrated dsRibbons^14^ [Fig. 1(d)], strongly suggesting that these conformations structurally have little in common. Third, fibers appear to contain both intramolecular and antiparallel β-sheets,^15^ and steric-zipper models are incompatible with both. The GK NMR structure also precludes Y39 from crosslinking. Finally, GK is incredibly rigid: GK is and can only adopt flat to RHH conformations with a much larger pitch than that has been observed for dsRibbons [Figs. 1(b) and 2 (Multimedia view)]. β-sheets comprised of non-glycine L-amino acids are inherently right-handed due primarily to intrasheet O/Cβ steric clashes.^24^

## RESULTS AND DISCUSSION

### β-helix homology model

The β-helix fold is often found in helical fibrils (e.g., bacteriophage P22 tailspike^25^) and is consistent with the αSyn fiber observations of parallel β-sheets that hydrogen bond parallel to the fiber axis. αSyn's central NAC domain has repeating hydrophobic motifs punctuated by glycine rich regions similar to those observed to form parallel sheets and turns in a β-helix. The β-helical structure of *Escherichia coli* glycosyltransferase LpxA^26^ has more sequence identity with the NAC domain than many other β-helical proteins [Fig. S1(c)]. Our β-helix NAC homology model was built from a pairwise aligned LpxA backbone [Fig. S1(d)] subjected to multiple rounds of manual fitting and Monte Carlo simulations (MCSs) with ROSETTA3.^27^ This yielded β-helix boundaries [Fig. S1(a)] and inter-residue interactions which unlike GK are in strong agreement with the two-stranded fibril distance restraints observed by NMR [Figs. 1(d) and 1(e)].

**FIG. 3.**
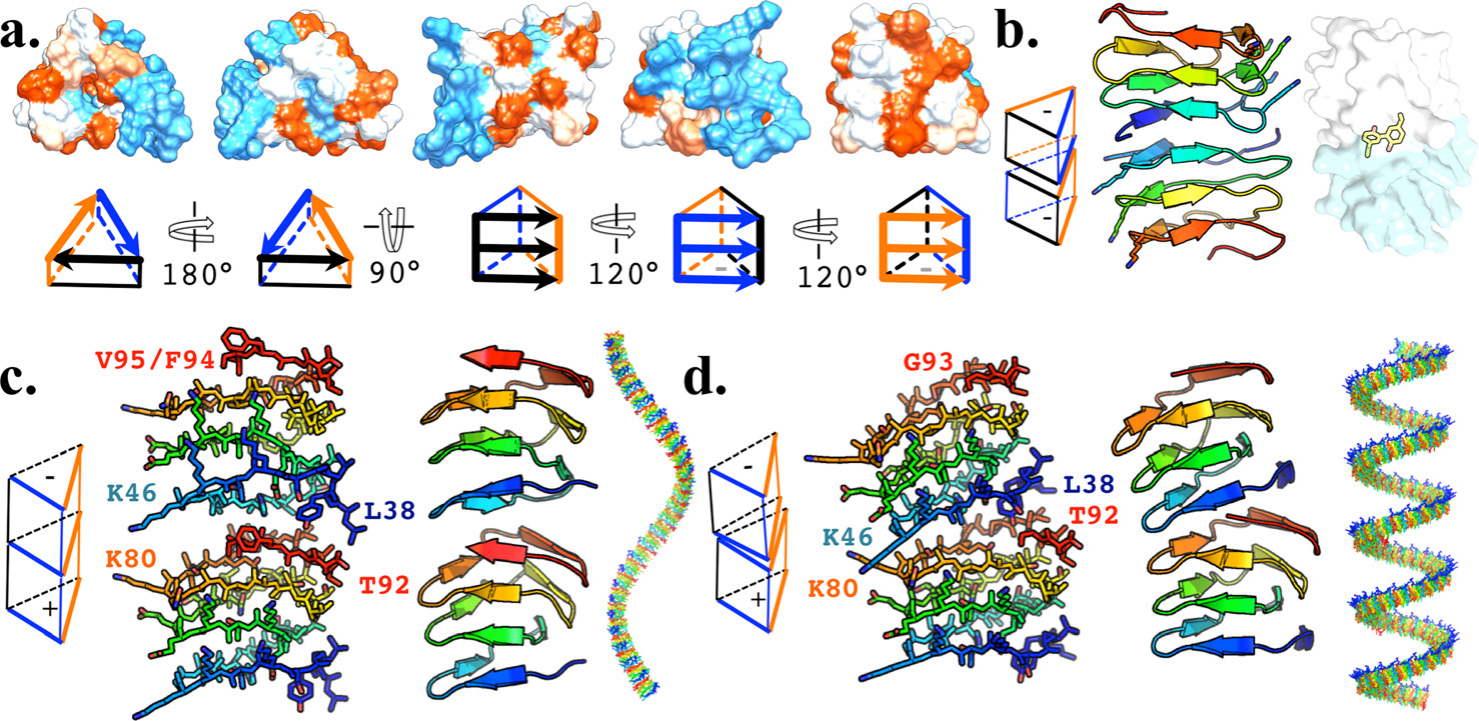
NAC β-helix. (a) NAC (residues 35–97) hydrophobicity surfaces displaying the CT base, NT base, amphipathic face (black in the schematic), alkaline face (blue in the schematic), and hydrophobic face (orange in the schematic). (b) Head-to-head dimerization between respective NT or CT bases would introduce antiparallel intramolecular β-sheets (cyan residues 48–50, left), which have been observed experimentally in non-fibrillar species, and is compatible with dityrosine crosslinking (yellow moiety, right). Head-to-tail oligomers are conductive to polymerization and can be modeled as LHH (c) or RHH (d) that matches observed periodicities by inclusion or exclusion of either the CT β-sheet including residues 94–95 or potentially NT β-sheets (not displayed).

Our NAC β-helix models compartmentalize five distinct surfaces with unique physiochemical characteristics [Fig. 3(a)]: an amphipathic face (∼residues 48–51 + 63–66 + 81–83), an alkaline face (∼residues 42–45 + 57–60 + 75–78 + 93–95), a hydrophobic face (∼residues 37–40 + 54–56 + 69–72 + 87–94) as well as NT and CT bases. Multiple units can interface by their NT/CT bases to extend intermolecular β-sheets. Rate-limiting dimerization of these β-helices may be facilitated by covalent crosslinking via Y39-Y39 di-tyrosine [Fig. 3(b)] and/or reactive oxidized compounds which are present at elevated levels during PD pathogenesis^28^ and have been shown to facilitate neurotoxic annular αSyn oligomerization *in vitro.*^29,30^ The alkaline face houses lysine residues positioned to crosslink with NT bases in a head-to-head [Fig. 3(b)] or NT to CT head-to-tail dimerization [Figs. 3(c) and 3(d)]. Head-to-tail dimerization is conducive to polymerization and could be seeded by head-to-head dimers. A head-to-head dimer is consistent with the non-fibrillar antiparallel β-sheet species observed by NMR^31^ and FTIR^15,32^ and the ∼1200 Å^2^ dimer collision cross-sections observed by mass spectrometry (MS)^33^ and could facilitate Y39-Y39 di-tyrosine crosslinking [Fig. 3(b)]. Such dimers are good candidates as stable prional species for intracellular transmission and have more favourable MCS Talaris energy function (TEF) scores than those of head-to-tail β-helix dimers, which are in turn much more favourable than GK dimers [Fig. S1(e)]. This is consistent with the GK cylinder irreversibly folding into dsRibbons,^13^ as the reverse operation is energetically unfavourable. As head-to-head dimers have accessible CTs for head-to-tail interaction, they may seed head-to-tail fibrillization.

### Mutagenic validation

**FIG. 4.**
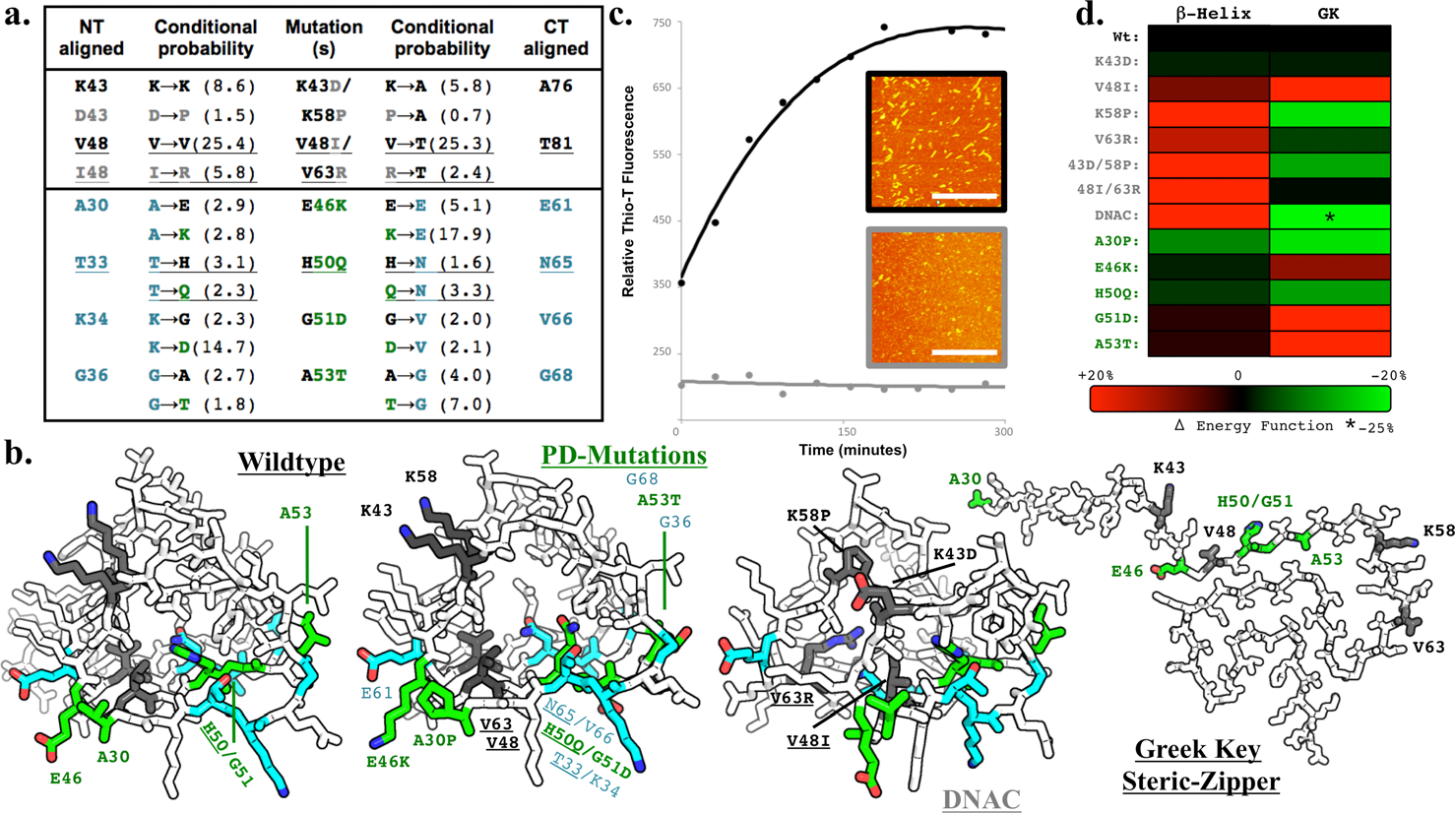
β-helix mutations. (a) β-helix conditional probabilities of amino acid alignment determined comprehensively from PDB deposited structures were used to design DNAC mutations (K43D/V48I/K58P/V63R, grey) to inhibit β-helix folding based on our homology model. The same β-helix conditional probabilities indicate that PD related human mutations E46K, H50Q, G51D, and A53T (green) are more likely to be aligned to the adjacent residues (cyan) in a β-helix than the wildtype αSyn residues are. (**b**) MCS topologies displaying DNAC mutations (grey), PD mutations (green), and PD mutation aligned residues (cyan). PD mutation β-helix stabilization is most easily observed with E46K, where K46 would stager the surface charge with E61 to increased stability. (c) Thioflavin-T binding fluorescence and AFM (scale bars 1 *μ*m) confirm that NAC (black) fibrillates rapidly and the DNAC mutant peptide (grey) does not, indicating that dsRibbons are not GK and may be β-helices with residues aligned as presented. (d) Heat map of the Rossetta3 Talaris energy function (inversely proportional to stability) change for each individual mutation indicates the synergistic destabilizing effect the four DNAC mutations have, which increases the stability of GK. Similarly, PD related mutations are predominantly more energetically favourable than wildtype to β-helix conformations, while being predominantly less favourable to a GK conformation.

To validate this β-helical NAC model, we used NAC constructs lacking the NT/CT passenger domains (αSyn residues 30–99) to focus on the NAC structure alone. AFM revealed that these truncated peptides can assemble to form fibers [Figs. 4 and S1(f)]. We designed mutations predicted to be specifically inhibitory to β-helix fibrillization and not GK steric zipper fibrillization. These mutations were designed using previously reported conditional probabilities,^34^ which were derived comprehensively from β-helix structures deposited in the PDB, to analyze intramolecular β-strand interactions and score probabilities of amino acid alignment, for both buried and exposed side chains (e.g., There is a 28% probability that surface exposed L residues contact K residues on an adjacent β-sheet but only 7% probability for the same alignment when the bulky side chains are buried within the β-helix).

Two residues we predicted to be exposed and aligned from the alkaline face were mutated (K43D/K58P), as were two buried residues from the amphipathic face (V48I/V63R) [Figs. 4(a) and 4(b)]. V48I/V63R reduces the probability of alignment buried within a β-helix from {V48/V63} = 25.4 to {I48/R63} = 5.8. Similarly, the residue we predict to be adjacent to V63R, T81, also had a reduction in β-helix alignment probability from {K48/A76} = 5.8 to P{P48/A76} = 0.7. The destabilized NAC^K43D/K58P/V48I/V63R^ quadruple mutant peptide (DNAC) has a >6-fold reduction in β-helix alignment probability. Thioflavin-T fibrillization assays and AFM [Fig. 4(c)] revealed that the truncated NAC constructs formed fibrils rapidly (K_M_ ∼ 75 min), whereas DNAC did not fibrillate within 48 h (only 8 h data shown). These mutations were predicted to have an energetically unfavourable alignment with β-helices^34^ but are all surface-exposed in a GK conformation [Fig. 4(c)].

This β-helix probability model can also rationalize the mechanisms of early onset PD associated with the familial mutations A30P, E46K, H50Q, G51D, and A53T. All these mutations reside on the amphipathic face, and with the exception of A30P, all increase the probability of β-helix alignment [Figs. 4(a) and 4(b)]. H50Q has the lowest increase to β-helix probability, with the pathogenic Q50 mutation being only twice as likely as H50 to be followed by adjacent buried residue N65. The acidic exposed side chain of D51 has six times the propensity of G51 to be aligned with the alkaline residue K34, with staggered surface charge topology to facilitate β-helical stability. In contrast, both G51D and A53T are buried in the GK steric-zipper, and these mutations would cause steric hindrance that would inhibit fibrillogenesis [Fig. 4(b)]. Although A30P precedes the NMR defined NAC β-sheets, P30 would present the NT passenger domain at a more acute angle, consistent with the observed A30P variant fiber morphology and assembly with faster initial fibrillation.^35^

Similarly, MCS predicted energy barriers to folding of a β-helix were less energetically favorable than wildtype for DNAC and more energetically favorable or very similar for human pathogenic mutations [Fig. 4(d)]. The same trends are not true for GK steric-zipper dimers, where the DNAC mutations were 25% more energetically favorable than wildtype GK.

These biophysical and computational mutagenic analyses indicate that these NAC fibrils and other pathogenic species are not GK and may be β-helices with side chains of V53/V63 buried and K43D/K58P exposed and aligned as modeled. Human PD-related pathogenic mutations might impart pathogenesis by lowering the energy barrier to the formation of the pathological β-helix fold.

### Supramolecular fiber structures

Unlike GK steric-zipper fibers, which are flat to mildly RHH, β-helix head-to-tail fibers can be modeled from LHH to RHH by minor adjustment to the intermolecular interface, e.g., respective inclusion or exclusion of a CT β-sheet [residues 93–95, Figs. 2 (Multimedia view), 3(c) and 3(d)]. Such a variable intermolecular interface is consistent with the variability of previously observed sheet boundaries by ssNMR [Fig. S1(a)^14,21,22^] in which the RHH fibers appear to have shorter β-sheets. Our RHH model has one less sheet on the alkaline face, resulting in ∼$\frac{1}{3}$ of the periodicity of the LHH model, which is congruent with the periodicities observed by AFM and EM.^17,19,20^

**FIG. 5.**
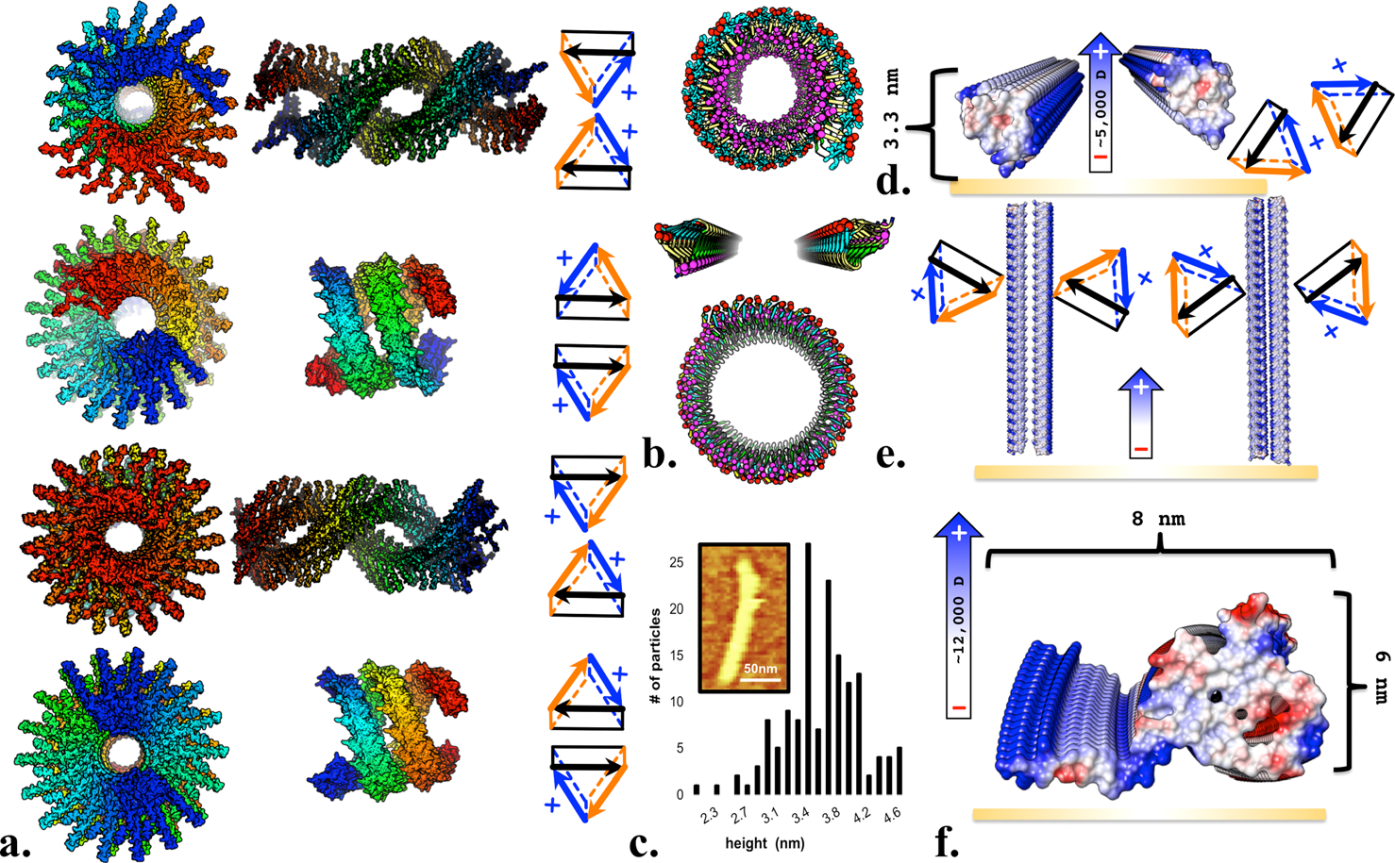
Fibril structure. (a) Four potential helical β-helix fiber configurations (2× 20mers) that are not sterically hindered by NT or CT, individual strands colored NT to CT, with schematics displaying orientations of the alkaline face (blue), amphipathic face (black), and hydrophobic faces. Top to bottom: LHH antiparallel; RHH antiparallel; LHH parallel and RHH parallel. (b) Axial views of NAC fibers displaying orientations of the alkaline face (cyan), its antipode turn (magenta spheres), the amphipathic face (green), and its antipode turn (red spheres). LHH (top) has the alkaline face's antipode turn (magenta) on the inner surface towards the fiber axis; straight fibers (middle) interface the alkaline face to the amphipathic face; and RHH (bottom) has the amphipathic face as the axial surface. (c) NAC fibrils grown on mica exhibited an average height of 3.5 nm, which is congruent with that of flat, antiparallel β-helix strands (d). Parallel strands (e) have dipoles parallel with the fiber axis and may be expected to grow away from the substrate, which was not observed, and single strand GK steric zippers are much larger than 3.5 nM in all dimensions. (d)–(f) 20-mer strands colored by the Coulombic surface, dipoles displayed in Debye.

We do not speculate with confidence about conformations for NT and CT passenger domains; however, both our LHH and RHH models can be modeled as dsRibbons with parallel or antiparallel strands without likely steric hindrance from the terminal domains [Fig. 5(a)]. These conformations would allow for NT/CT intercalation akin to what has been observed by paramagnetic relaxation enhancement (PRE) experiments,^36^ which might stabilize the fairly regular periodicity often observed. From our model, the outer equatorial surface of the LHH NAC fiber is the alkaline face of the β-helix with the inner axial surface representing the antipode turn to the alkaline face, an orthogonal configuration with an interior amphipathic face and its antipode turn, and the interior is modeled for RHH [Fig. 5(b)].

Tapping mode AFM reveals that our NAC constructs (residues 30–99) grow as straight, flat ribbons on a mica substrate [Fig. 5(c)], as has been observed previously for full length αSyn grown on mica.^16^ The observed average height of 3.5 nm closely matches the height of β-helix fiber models [Figs. 5(d) and 5(e)] and excludes the GK steric-zipper, which is considerably larger than the observed height in every dimension, even as a single strand [Fig. 5(f)].

If potential fiber helicity is continuous from RHH to LHH, as we have modeled [Fig. 2 (Multimedia view)], then the flat antiparallel strands' intermediate fiber interface would likely be oriented from the amphipathic face to the alkaline-face [Figs. 5(b) and 5(d)]. This configuration would not likely exclude the terminal domains by steric hindrance, and any antiparallel fiber configuration would have a strong additive macrodipole orthogonal to the fiber axis (∼5000 Debye for a 20-mer as calculated using the Weizmann Protein Dipole Moments Server^37^). An orthogonal dipole is consistent with the fiber orientation observed by AFM [Figs. 5(c) and S1(f)] as all fibers were grew parallel to the mica substrate, which is positively charged. A parallel fiber configuration would render all charges perpendicular to the fiber axis to be diametrically opposed. In such an orientation, a comparatively weak dipole parallel to the fiber axis would exist and growth away from the substrate would be expected [Fig. 5(e)].

### Annular models

**FIG. 6.**
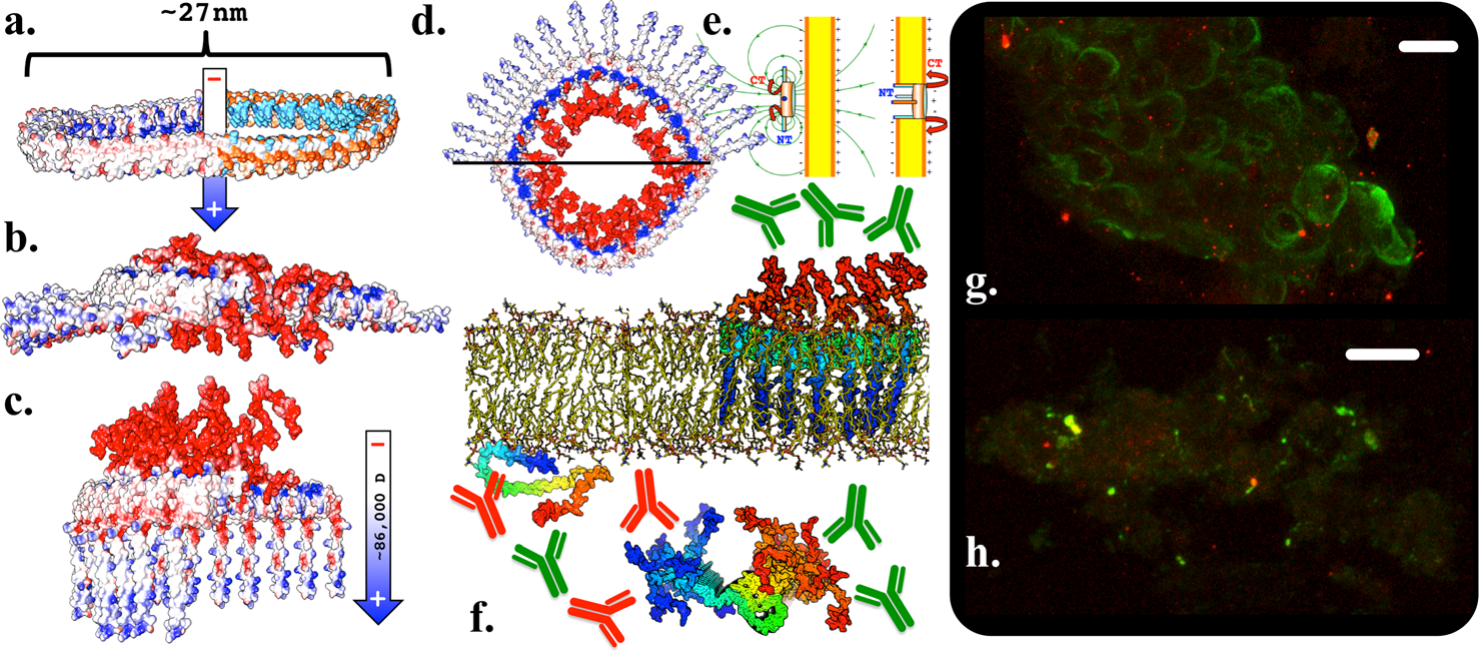
Annulus models and epitope mapping: (a) Coulombic and hydrophobic surfaces displaying a NAC β-helix toroid 36mer backbone with a hydrophilic inner ring of alkaline faces amenable to a pore. Half toroid structures displaying the proposed peripheral (b) and integral (c) conformations. Amphipathic NT α-helices initially orient acidic CT tails away from the membrane [(d) top] and then contribute to the transmembrane pore itself when the CT domains are extended to puncture the polarized membrane [(d) bottom]. (e) Approximate magnetic field lines of a toroid dipole, which could facilitate polarized membrane insertion. (f) NT to CT colored structures, indicating integral αSyn pore NT inaccessibility and potential high avidity for immunostaining (top), whereas both NT and CT epitopes are accessible for monomeric and fibular structures (bottom). Comparatively intact (g) and necrotic (h) cells derived from rat midbrain neurons transfected to overexpress αSyn^A53T^ were immunostained with synuclein NT (red, EP1646Y epitope 1–30) and CT (green, LB509 epitope residues 115–122) specific antibodies. Scale bars represent 10 *μ*m, and each pixel is ∼70 nm. Diffuse intracellular staining of each epitope is observable for each epitope, with stronger CT staining at intact cell membranes (g) where the lipid-binding NT domains are less accessible. Necrotic cells (f) display CT foci at the extracellular membrane periphery consistent with high avidity breached transmembrane pores.

Cytotoxic pore-forming^38^ annular species with heights of 3–6 nm and diameters of 24–91 nm observed by AFM,^39^ EM,^40^ and SAXS^41^ have been shown to nucleate two-stranded fibrils *in vitro*,^39^ and conversely, annuli have been observed to writhe and splinter off from supercoiled dsRibbons,^42,43^ indicating that the species exist in equilibrium. Both dsRibbons and annuli are similarly susceptible to proteinase K digestion,^42^ indicative of considerable conformational coincidence not shared with ssFiber steric-zippers. Cytotoxic assemblies require α-helical NT;^44^ however, NAC β-helices modeled into a toroid with the appropriate diameters have properties consistent with forming a pore: the β-helix alkaline faces establish a hydrophilic inner ring, the hydrophobic faces form the outside, and a strong macrodipole runs perpendicular to the ring [Fig. 6(a)]. The inconsistent sizes of these assemblies indicate that perfectly symmetrical annuli are unlikely *in vivo* but may still be representative of underlying features of *in vivo* β-helix arcs occluded in aggregation.

Pre-cytotoxic aggregates retain and require the amphipathic NT helix observed in vesicle-bound monomeric αSyn,^44^ which aid in orientation and folding peripheral to membranes. This is consistent with our β-helical model as NT is exposed equatorially in our pre-cytotoxic membrane peripheral model and, as it is amphipathic, may contribute to the transmembrane cytotoxic pore in an axial conformation [Figs. 6(b)–6(d)].

**FIG. 7.**
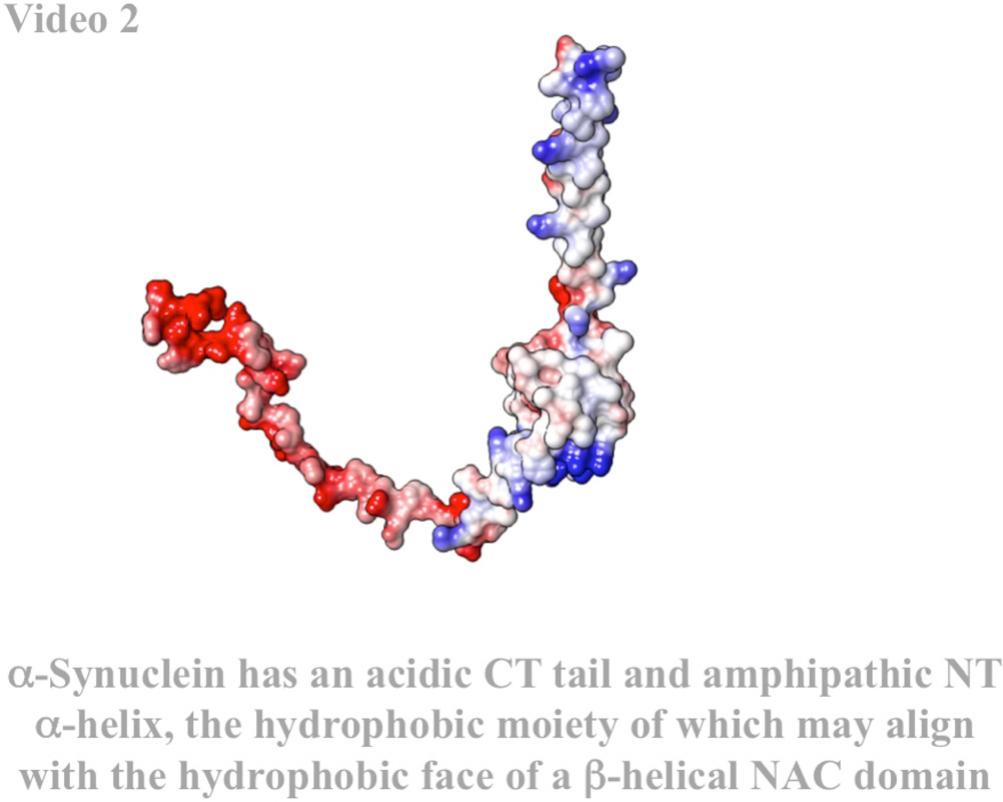
Proposed mechanism of αSyn toroid cytotoxicity. Toroids and arcs are natural polymerization endpoints to membrane peripheral αSyn docked by amphipathic NT α-helices. The same NT-helices may extend and contribute to a pore by electromotive force of CT tail discharge and extension to the extracellular face of a polarized membrane. Multimedia view: https://doi.org/10.1063/1.5023460.210.1063/1.5023460.2

Axial acidic CT tails would contribute significantly to the toroidal dipole [∼86 000 Debye, field lines approximated in Fig. 6(e)]. Polarized membranes such as presynaptic membranes and/or alkaline seed structures might electrostatically facilitate folding of these pathogenic species, and the resultant strong dipole would facilitate membrane insertion into polarized membranes [Figs. 6(e) and 6(f)]. CT tails might drive membrane insertion by discharging to the cationic face to breach the polarized membrane [Fig. 7 (Multimedia view)] in a mechanism not unlike the membrane attack complex of the complement system, which is also a transmembrane β-sheet toroidal pore of comparable size.^45^ Our membrane pre-cytotoxic peripheral conformation, including helical NT domains, is consistent with PRE analysis of lipid imbedded residues of pre-cytotoxic oligomers.^44^

**FIG. 8.**
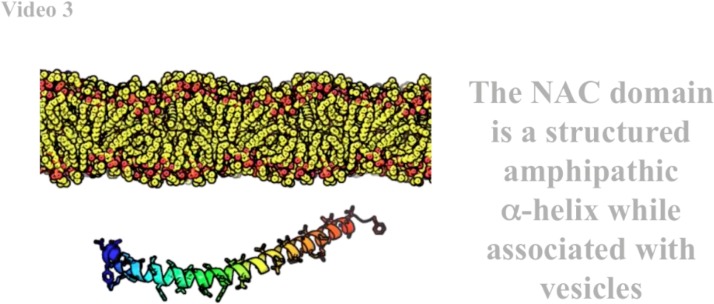
β-helical NAC pools provide pathogenic equilibrium. The NAC domain is α-helical while associated with vesicles and disordered in solution. A β-helix is a comparatively stable monomeric conformation. There is a favourable energetic pathway for GK steric-zippers to fold into β-helices, while the reverse operation is unfavourable. As stable β-helix dimer species are formed, they can seed larger ordered complexes such as the annular cytotoxic pore. Annular species have been demonstrated to seed fibers, which in turn have been demonstrated to seed or alternatively writhe and splinter off annular species. The annulus serves as a portal for intercellular transmission of stable dimer prional species, which themselves may fragment off from larger β-helical complexes. Multimedia view: https://doi.org/10.1063/1.5023460.310.1063/1.5023460.3

*Ex vivo* evidence for this general configuration can be seen in neurodegenerative αSyn^A53T^ transfected rat midbrain^46^ cells [Figs. 6(g) and 6(h)] immunostained with αSyn antibodies specific to the CT (green, LB509: epitope residues 115–122) and NT (red, EP1646Y: immunogen residues 1–30). Comparatively intact cells [Fig. 6(e)] display diffuse intracellular immunostaining for both antibodies binding to cytoplasmic synuclein, with CT immunostaining prominent at the cell membranes where the hydrophobic NT domains are less accessible. Necrotic cells [Fig. 6(f)] have foci of accessible CT not colocalized with accessible NT. Given their dimensions (70–700 nm), these features may represent high avidity transmembrane pores with CT-tails exposed on the cell surface.

## CONCLUSIONS

Pathogenic species such as antiparallel dimers, two-stranded fibers, and neurotoxic annuli have already been demonstrated to exist in equilibrium. A unified β-helix conformational pool for this pathogenic conformational equilibrium [Figs. 7 (Multimedia view) and 8] is consistent with parallel discontinuous β-sheets oriented orthogonal to the fiber axis and gives a structural basis for the observed NAC fiber dimensions, protease sensitivity, and mechanisms of human pathogenic mutations, as well as providing a possible mechanism for cytotoxicity. We hope that our models may be of use for structure-based design of novel therapeutics targeting pathogenic α-synuclein conformers.

## METHODS

NAC (residues 30–99) and DNAC (residues 30–99^K43D;V48I;K58P;V63R^) peptides were synthesized by Biomatik^®^ (Cambridge, Canada). Antibodies LB509 and EP1646Y were purchased from Abcam^®^ (Cambridge, UK). When not explicitly stated, other reagents were obtained from Sigma-Aldrich^®^ (St Louis, MO).

### Thioflavin-T assays

Fluorescence was measured using a Synergy NEO plate reader (BioTek^®^, Winooski, VT) for 72 h with orbital shaking in a 96 well plate immediately after protein suspension and 0.22 *μ*m filtration (EMD Millipore^®^, Darmstadt, Germany). 100 *μ*l of reaction mixture contained 10 *μ*M protein, 10 *μ*M Thioflavin-T, 10 mM HEPES (pH 7.4), and 150 mM NaCl in water.

### Atomic force microscopy

The protein samples were suspended to 10 *μ*M in 10 mM HEPES containing 150 mM NaCl at pH 7.4 and immediately introduced to the fluid cell. Fibril formation was imaged and monitored by fluid tapping mode atomic force microscopy performed at room temperature until the process stabilized. All images were acquired in the fluid tapping mode on a Nanoscope IIIA Multimode AFM (Bruker Nanosystems, Santa Barbara, CA) equipped with a J scanner (maximum lateral scan area 125 × 125 *μ*m and maximum vertical scan 5 *μ*m), using SNL-10 tip C (Bruker, Camarillo, CA) using the Nanoscope software version 5.12r3. A glass fluid cell was sealed with a silicone O-ring against a freshly cleaved mica substrate. Both AFM probes and fluid cells were exposed to UV light for 15 min to remove possible organic contaminants. AFM images were collected at a resolution of 512 × 512 pixels at a scan rate of 1 Hz using a drive frequency of 8–10 kHz. The height images were processed using Nanoscope software version 5.12r3. The length and width values of the fibers were determined using ImageJ version 1.46r using the following sequence of image processing steps: (1) despeckle; (2) adjust threshold; (3) particle analysis using the fit ellipse measurement with the size from 0 to infinity and circularity from 0.0 to 1.0. The height values were determined by particle analysis in NanoScope Analysis software version 1.50 (Bruker, Santa Barbara, CA) without tip deconvolution.

### β-helical model generation and Monte Carlo simulations

Local sequence alignments of the αSyn NAC and several β-helices with solved structures found high sequence identity with *E. coli* glycosyltransferase LpxA^26^ (PDB 2AQ9). LpxA residues 151–180 were pair-wise aligned to αSyn residues 69–98 [Fig. S1(d)] as a starting model for manual mutagenesis, and repeating units of this starting model were used to model residues 30–68 with multiple rounds of fitting with SETOR^47^ and COOT^48^ with intermittent energy gradient minimization and deterministic MCS without explicit solvent in ROSETTA3^27^ (default ROSETTA energy function 2015)^49^ and Foldit stand-alone^50^ until simulations reached steady states. Additional geometry regularization and energy minimization were achieved with COOT,^48^ Chimera,^51^ and the PHENIX software suites.^52^ Once a satisfactory monomeric model was achieved (TEF < 0), similar rounds of refinement were applied to small multimers.

Large multimers (>6 molecules) were assembled from smaller multimers without additional refinement by alignment in SETOR and/or Pymol.^53^ For the annulus, a nearly circular RHH starting model hexamer was manually adjusted to be in plane at a 60° angle, subjected to refinement rounds as above with the 1st and 6th chains static (TEF-24.3), and then overlapped into a toroidal 36mer. Where NT/CT domains are displayed, they were aligned at appropriate residues directly from PDB(s) 2KKW and/or 2N0A without additional refinement. These domains are not modeled with confidence and are only displayed for illustrative purposes.

**FIG. 9.**
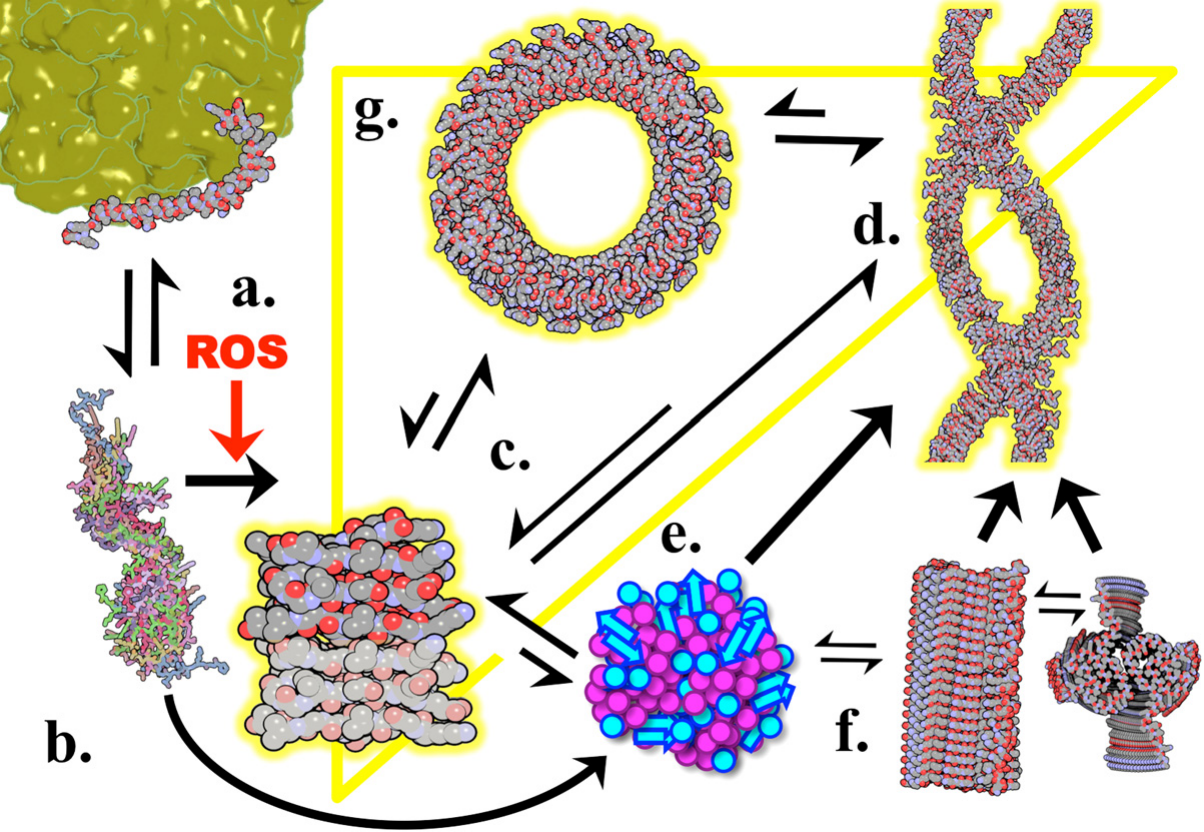
αSyn conformer equilibrium. αSyn NAC residues 30–99 are predominantly amphipathic α-helices when vesicle bound (a) and highly disordered in solution (b). Rate limiting αSyn dimerization (c) can be induced with reactive oxygen species (ROS) and includes covalent dimers and species with a β-sheet structure both parallel and antiparallel, which are compatible with a β-helix. Prional β-helical dimers may polymerize directly into β-helical dsRibbons (d) or form metastable aggregates (e), which in turn may seed dsRibbons or steric-zipper cylinders (f). Single or double stranded cylinders can be consumed to seed dsRibbons, but the reverse operation is energetically unfavourable and not observed. β-helical species (yellow triangle) can directly form cytotoxic pores (g), and the reverse operations have also been observed.

Hydrophobicity surfaces and Coulombic surfaces were calculated and rendered with Chimera, Fig. 9 was rendered in QuteMol,^54^ and other structures were rendered with Pymol. Lipid structures, which are included for illustrative purposes only and not used in MCS, were derived from coordinates provided by Peter Tieleman's Biocomputing Group at the University of Calgary^55^ and PDB 2MLR.^56^ Protein macrodipole values were estimated using the Weizmann Protein Dipole Moments Server.^37^ Identities for exposed residue mutagenesis predicted to be inhibitory to a β-helix were inferred from BETAWRAP^34^ conditional probabilities, which were comprehensively derived from all β-helix structures deposited in the PDB at the time of publishing. Talaris energy functions (TEFs) are inversely proportional to conformer stability and were calculated for NAC models using energy gradient minimization without explicit solvent in ROSETTA3.^27^ The GK monomer TEF is reported as deposited without energy gradient minimization, which would cause adoption of a globular conformation. GK mutant dimer TEFs were minimized with static terminal residues to prevent more favourable non-GK folding.

### Immunostaining and confocal imaging

Rat transfection with αSyn^A53T^ and preparation of 40 *μ*m cryosections have been described in detail elsewhere.^46^ Animals were housed in pairs in a temperature controlled environment, kept on regular 12 h light/dark cycles, and allowed food and water *ad libitum*. All procedures were conducted under permit 1738, which received local IACUC approval (University Health Network). Midbrain regions including the *substantia nigra* were excised from cryosections, minced with a razor, and subjected to 30 min of partial disintegration by Collagenase and Dispase II digestion with nutation and pipette trituration. Partially disintegrated cells were applied to a glass slide, incubated at 37 °C for 2 h to facilitate adhesion, fixed with 4% paraformaldehyde overnight at 4 °C, washed 5× with phosphate buffered saline (PBS), blocked for 2 h at room temperature in 10% normal goat serum with 1% bovine serum albumin in PBS, and incubated overnight at 4 °C with primary antibodies (LB509 and EP1646Y) diluted 1:5000 in blocking solution. Samples were washed 3× for 15 min with PBS, incubated for 2 h at room temperature with secondary antibodies anti-rabbit Alexa Fluor^®^ 568 and anti-mouse Alexa Fluor 488 (Thermo Fisher Scientific, Fair Lawn, NJ), diluted 1:5000 in blocking solution, washed 3× for 15 min each with PBS, mounted with signaling stain mounting medium (Cell Signaling Technology, Beverly, MA), and imaged with a Zeiss LSM700 Confocal microscope using a 63× oil immersion lens (Carl Zeiss Inc., Thornwood, NJ).

## SUPPLEMENTARY MATERIAL

See supplementary material for additional Fig. S1.
